# Transmembrane Protein 230 Mediates a Poly(ADP-ribose) Polymerase-1-Linked Apoptosis

**DOI:** 10.3389/fnagi.2020.00235

**Published:** 2020-08-07

**Authors:** Xiaobo Wang, Tengteng Wu, Jinru Zhang, Gongbo Guo, XiaoFei He, Zhong Pei, Zhaohui Liu, Chun-feng Liu, Christopher A. Ross, Wanli W. Smith

**Affiliations:** ^1^Institute of Neuroscience, Soochow University Medical College (SUMC), Suzhou, China; ^2^Department of Psychiatry and Behavioral Sciences, Johns Hopkins University School of Medicine, Baltimore, MD, United States; ^3^Department of Neurology, The First Affiliated Hospital, Sun Yat-sen University, Guangzhou, China; ^4^Department of Neurology, The Second Affiliated Hospital of Soochow University, Suzhou, China; ^5^Department of Human Anatomy and Cytoneurobiology, School of Biology and Basic Medical Sciences, Soochow University Medical College (SUMC), Suzhou, China

**Keywords:** Parkinson’s disease, TMEM230, reactive oxygen species, PARP1, apoptosis

## Abstract

Mutations in transmembrane protein 230 (TMEM230) gene are suggested to be associated with the autosomal dominant Parkinson’s disease (PD) with typical movement disorders and Lewy body pathology. However, the normal functions and the pathological roles of TMEM230 are not clear. In this study, we used TMEM230 isoform II constructs including wild-type (WT) and four reported PD-linked mutation constructs (Y92C, R141L, 184Wext*5, and 184PGext*5). Ectopic expression of WT and PD-linked mutant TMEM230 variants in cultured cells dramatically induced apoptotic cell death compared with that of vector control cells. Mutant TMEM230 caused cell toxicity at an increased severity than WT TMEM230. Moreover, expression of TMEM230 increased mitochondrial reactive oxygen species (ROS) levels, decreased cellular ATP, activated caspase 3/7, and increased poly(ADP-ribose) polymerase-1 (PARP1) cleavage. Treatment with *N*-acetylcysteine (NAC; an ROS scavenger) or Z-VAD-FMK (a caspase inhibitor) significantly attenuated TMEM230-induced apoptosis in both cultured cells and primary neurons. Our results indicated that TMEM230 mediated a PARP1-linked apoptotic cell death pathway. These findings not only provide the novel insight into the biological roles of TMEM230 in the PARP1-linked pathway but also provide a TMEM230-induced cell death mechanism underlying PD pathogenesis.

## Introduction

Parkinson’s disease (PD) is a common neurodegenerative disease with movement disorder. The clinical features of PD include tremors, muscle rigidity, bradykinesia, and postural instability in movements due to selective loss of dopaminergic neurons in the brain (Shi et al., 2017). PD affects about 1% of individuals aged over 60 years and about 4–5% of those aged over 85 years (Lew, 2007). The molecular pathogenesis is not completely understood (Yarnall et al., 2012). Mutations in several genes have been identified to be associated with familial form of PD, including α-synuclein, LRRK2, parkin, PINK1, DJ-1, VPS35, and recently identified transmembrane protein 230 (TMEM230; Williams-Gray and Worth, 2016).

Mutations in TMEM230 gene have been reported initially to play a pathogenic role in a rare autosomal dominant PD with typical movement disorders and Lewy body pathology (Deng et al., 2016). Mutations in TMEM230 were first identified to cause familial PD by Deng’s group (Deng et al., 2016). Recently, studies by other groups suggest that TMEM230 is more likely to be a risk-associated gene for PD (Giri et al., 2017; Quadri et al., 2017). TMEM230 gene encodes two isoforms of TMEM230 proteins, with 183 amino acids (I) and 120 amino acids (II; Deng et al., 2016). Isoform-2 accounts for ~95% of total protein isoforms in humans (Deng et al., 2016). Isoform-2 contains two transmembrane segments, with N-terminal and C-terminal regions exposed to the cytosol (Deng et al., 2016). *TMEM230* mRNA is highly expressed in many tissues, including the midbrain, neocortex, cerebellum, and spinal cord (Deng et al., 2016). TMEM230 appears to localize in vesicle structures (e.g., synaptic vesicles) in neurons (Deng et al., 2016).

There are four PD-linked TMEM230 mutations (Y92C, R141L, 184Wext*5, and 184PGext*5; Deng et al., 2016). 184Wext*5 mutation replaces the stop codon with tryptophan and adds five additional amino acids (HPPHS). 184PGext*5 mutation changes the stop codon to proline and adds glycine with five additional amino acids (HPPHS). R141L, Y92C, and 184Wext*5 are detected in North American PD cases, whereas 184PGext*5 is identified in seven Chinese PD families. Y92C and 184PGext*5 mutations display incomplete penetrance (Deng et al., 2016). TMEM230 mutation cases exhibit late-onset, good-response to levodopa and display clinical features including rigidity, rest tremor, bradykinesia, dementia, neuronal loss in the substantia nigra and nucleus basalis, and presence of Lewy bodies (Deng et al., 2016).

The functions of TMEM230 are not clear. Increasing evidence suggests that TMEM230 may be involved in multiple cellular processes that are linked to PD pathogenesis (Calhoun et al., 1996; Deng et al., 2016; Kim et al., 2016). TMEM230 is co-localized with synaptic vesicle trafficking associated proteins including synaptophysin, vesicle-associated membrane protein 2 (VAMP2), vesicular monoamine transporter 2 (VMAT2), and VPS 35 (Calhoun et al., 1996; Deng et al., 2016). Knockdown or expression of PD-linked mutant TMEM230 induces a synaptic vesicle trafficking deficit (Deng et al., 2016) and abnormalities in trans-Golgi network secretion (Kim et al., 2016). TMEM230 is co-immunostained with Rab 5a, Rab11, and Rab 8a, which are involved in endosomal recycling and trafficking, vesicle secretion, and autophagy (Kim et al., 2016). Some of Rab family proteins can be phosphorylated by LRRK2, which may lead to TMEM230 interplay with LRRK2 *via* Rab-linked pathways. Moreover, a study showed that expression of mutant TMEM230 increases a-synuclein levels (Deng et al., 2016) and induces puncta or aggregates in SN4741 cells (Nam et al., 2018). Given that the pathological roles of mutant TMEM230 are largely unknown, further studying TMEM230 could provide a better understanding of the pathogenesis and identify novel targets for intervention.

In this study, we used TMEM230 isoform II cDNA construct, along with four reported PD-linked mutation constructs (Y92C, R141L, 184Wext*5, and 184PGext*5) to determine the roles of TMEM230. Our results showed that TMEM230 plays a critical role in the poly(ADP-ribose) polymerase-1 (PARP1)-linked cell death pathway. PARP1 is an ADP-ribosylating enzyme essential for initiating various forms of DNA repair. In the pathological condition, extensive DNA damage in cells results in PARP1 cleavage (inactive), preventing DNA repair and thereby leading to cell apoptosis or necrosis (Swindall et al., 2013; Morales et al., 2014). Abnormal PARP1 cleavage has been implicated in many diseases, such as the neurodegenerative diseases including PD (Lee et al., 2013, 2019). Our studies not only provide novel insight into the biological roles of TMEM230 in the PARP1-linked pathway but also provide a TMEM230-induced cell death mechanism underlying PD-linked pathogenesis.

## Materials and Methods

### Reagents

Antibodies were from various vendors and used for this study including rabbit anti-TMEM230 (Sigma, HPA009078), rabbit anti-PARP (CST, 9532), rabbit anti-cleaved PARP (CST, 9541), mouse anti-c-myc (Roche, 1166723001), ECL goat anti-rabbit IgG (GE Healthcare, NA934V), and ECL goat anti-mouse IgG (GE Healthcare, NA931V). Z-VAD-FMK was from Santa Cruz. *N*-Acetylcysteine (NAC) was from Sigma. Trypan blue dye was from Gibco.

### Cell Culture, Plasmids, and Transfection

Human embryonic kidney HEK293T cells were from American Type Culture Collection (ATCC; Manassas, VA, USA) and were grown in Dulbecco’s modified Eagle medium (DMEM; Gibco) with 10% heat-inactive fetal bovine serum (FBS; Gibco) and 1% penicillin–streptomycin (Gibco) in a humidified 5% CO_2_ atmosphere at 37°C. Wild-type (WT) TMEM230 cDNA construct with Myc tag was from Addgene. Four PD-linked mutant (Y92C, R241L, 184Wext*5, and 184PGext*5) constructs with Myc tag were generated by Zhong Pei’s group. TMEM230 constructs were transfected into HEK293T cells using Lipofectamine PLUS (Invitrogen), according to the manufacturer’s protocol. Fifty micromolar of Z-VAD-FMK and 1 mM of NAC was added post transfection for 36 h.

### Cell Toxicity Assays

Trypan blue assays were used to detect living (bright) and dead cells (blue) at 24–48 h post transfection as described previously (Smith et al., 2005). Cell viability was calculated by a ratio of living cells/total cells (living plus dead). Hoechst 33342 nuclei staining was used to detect apoptotic cells with highly condensed and fragmented nuclei under fluorescent microscopy (Wei et al., 2002). Hoechst 33342 (Invitrogen) was added for 5 min prior to image taking and nuclei accounting. The counting of the apoptotic cells was performed by an investigator who was blind to experimental conditions.

### Measurement of Mitochondrial Reactive Oxygen Species

HEK293T cells were subjected to mitochondria superoxide detection after transfection for 24 or 48 h as described previously (Liu et al., 2013). Briefly, MitoSOX Red and MitoTracker Green were diluted to a concentration of 5 and 1 μM, respectively, in 0.1% dimethyl sulfoxide (DMSO), according to protocol (Invitrogen). Cells were incubated with MitoSOX and MitoTracker for 20 min at 37°C, and then unused free MitoSox was washed away using phosphate-buffered saline (PBS). Fluorescent images were captured under fluorescent microscopy image system with identical exposure settings (Carl Zeiss). The density of MitoSOX (Red) was quantified by NIH image software to represent the levels of mitochondrial superoxide. MitoTracker was used to confirm the localization of MitoSOX to mitochondria. Hoechst 33342 (blue) was added for nuclei staining.

### Cellular ATP Assays

Cellular ATP levels were measured using an ATP Determination Kit (A22066) from Invitrogen, according to manufacturer’s protocol. This is a bioluminescence assay to measure ATP with recombinant firefly luciferase and its substrate D-luciferin, based on luciferase’s absolute requirement for ATP in producing light (emission maximum ~560 nm at pH 7.8). Briefly, cell lysate (50 μg/per sample) was added with reaction solution (0.1 M of DTT, 10 mM of D-luciferin, and 2.5 μg of firefly luciferase) into 96-well solid white polystyrene microplates (Corning 3912). The luminescence of each sample was measured at ~560 nm by using a luminometer (GloMax, Promega).

### Immunofluorescence

Cells were fixed with 4% paraformaldehyde in PBS for 30 min, permeabilized with 0.2% Triton X-100 (Sigma) for 15 min, and blocked with 5% normal goat serum for 1 h as described previously (Liu et al., 2016). Anti-myc (1:300 dilution) or anti-TMEM230 (1:100 dilution) antibodies were used as primary antibodies followed by incubation with secondary detection antibodies (anti-rabbit Alexa-568 or anti-mouse Alexa-488 antibodies). Nuclei were stained with Hoechst 33342.

### Western Blot Analysis

HEK293T cells expressing TMEM230 that were treated with either vehicle or inhibitors were harvested using lysis buffer (Cell Signaling). The resulting lysates were subjected to protein assays. An aliquot of 30 μg of protein from each sample was resolved on 4–12% NuPAGE Bis/Tris gels and transferred onto polyvinylidene difluoride membranes (Millipore). After being washed with TBST (TBS and 0.05% Tween-20) buffer, the membranes were blocked with 4% milk for 1 h and then incubated with various primary antibodies: anti-TMEM230 (1:500 dilution), anti-myc (1:1,000 dilution), rabbit anti-PARP (1:1,000 dilution), and rabbit anti-cleaved PARP (1:1,000 dilution), followed by horseradish peroxidase (HRP)-conjugated secondary antibodies: goat anti-rabbit IgG (1:5,000 dilution), and goat anti-mouse IgG (1:5,000 dilution). Immunoblot signals were detected by ECL enhanced chemiluminescence reagents (PerkinElmer).

### Caspase 3/7 Detection

Nexcelom ViaStain™ Live Caspase 3/7 Detection kit (CSK-V0003-1, Nexcelom) was used to detect caspase 3/7 activities in live cells, according to manufacturer’s protocol. The principle of this no-wash assay is that the NucView™ reagent with a nucleic acid-binding dye linked to a fluorescent probe is attached to a four-amino acid peptide sequence DEVD (Asp-Glu-Val-Asp), forming a cell membrane-permeable DEVD–DNA complex. When the nucleic-acid dye is linked to the DEVD peptide sequence, the dye is unable to bind to DNA and remains non-fluorescent. During apoptosis, activated caspase 3/7 cleaves the DEVD–DNA dye complex to release the high-affinity DNA dye, which translocates to the nucleus and binds to the DNA, producing a bright green fluorescent signal. Cells were transfected with vector or TMEM230 constructs for 24–48 h and then incubated with NucView™ reagent for 30 min. The green fluorescence was read by using Celigo fluorescent reader.

### Primary Neuron Cultures and Viability Assay

Primary mouse cortical neurons were cultured from E15 to E17 embryos as described previously (Eddings et al., 2019), in neurobasal medium with added 2% B27, 1% PenStrep, and 2 mM of GlutaMAX. At day *in vitro* (DIV) 5, neurons were co-transfected with TMEM230 plasmids and green fluorescent protein (GFP) at a ratio of 10:1 using Lipofectamine 2000 (Thermo Fisher Scientific, Waltham, MA, USA) as described previously (Eddings et al., 2019). The transfection rate was determined to be around 5%. There was about 95% GFP positive neurons expressing TMEM230.

Neuron viability assays were performed as described previously with slight modifications (Eddings et al., 2019), Briefly, post transfection 46 h, neurons expressing GFP were automatically imaged for 2 h with 20-min interval on a Zeiss Axiovert 200 inverted microscope. Neuronal morphology was blindly quantified using ImageJ software. Manual classification of cell death was performed using morphological criteria. Dead cells were defined as they displayed loss of neuronal projection disappearance, presence of soma fragmentation, or soma shape change from oval to completely circular. An arbitrary morphological score was assigned as either 100 for alive cells or 0 for dead cells in each frame. The mean morphological score of all the cells was calculated. Survival analysis was performed using a Gehan–Breslow test to determine a statistical difference between groups, followed by an all pairwise multiple comparison (Holm–Sidak method) to identify the differences between groups.

### Statistical Analysis

Quantitative data represent arithmetic means ± SEM from three separate experiments. For all biochemistry experiments, there were three parallel reactions for each sample. Statistically significant differences among groups were analyzed by ANOVA by using the GraphPad Prism 5 software. A *p*-value < 0.05 was considered statistically significant.

## Results

### Ectopic Expression of TMEM230 Induced Apoptotic Cell Death *in vitro*

**Figure 1:**
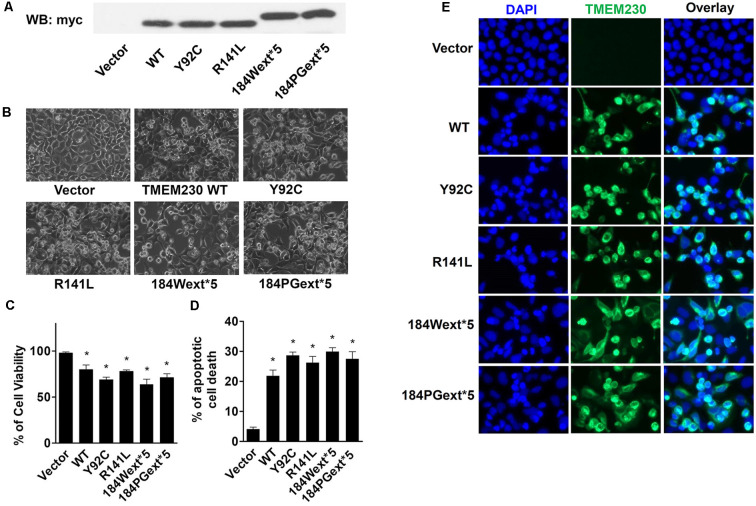
Ectopic expression of transmembrane protein 230 (TMEM230) induced apoptotic cell death *in vitro*. HEK293T cells were transfected with vector or various TMEM230 constructs as indicated for 48 h. **(A)** Expression of exogenous Myc tagged TMEM230 variants in HEK293T cells by western blot analysis using anti-Myc antibodies. **(B)** Representative cell images under light microscope. **(C)** Trypan blue assays were used to count living (bright) and dead (blue) cells under light microscope. Cell viability was calculated. **(D)** Apoptotic cells were counted in each group using Hoechst 33342 staining. **(E)** Representative fluorescent images. Cells were immunostained using anti-Myc antibodies. *p* < 0.05 by ANOVA, *vs. cells expressing vector.

Human isoform II TMEM230 constructs (WT, Y92C, R141L, 184Wext*5, and 184PGext*5) were transfected into HEK293T cells for 48 h. Expression of WT or mutant TMEM230 (Figure 1A) displayed a shrunken morphology with rounded cell bodies and retracted processes compared with that of vector control cells (Figure 1B). Trypan blue assays were used to measure cell viability. To our surprise, expression of WT alone caused cell toxicity compared with that of vector control cells (Figure 1C). Moreover, PD-linked TMEM230 mutants cause more severe cell death.

Apoptotic cells can be detected by Hoechst 33342 fluorescent labeling with highly condensed and fragmented nuclei (Wei et al., 2002). To further study TMEM230-induced cell death, we used Hoechst 33342 nuclei staining to identify high condensation and fragmented nuclei (sick or dead apoptotic cells). Both WT and mutant TMEM230 caused apoptosis, whereas PD-linked mutant caused more apoptotic cell death (Figures 1D,E). These results suggest that TMEM230 is critical for controlling apoptotic cell death. All four PD-linked mutants caused approximately similar cell toxicity. We only selected two mutants (184Wext*5 or 184PGext*5) to perform the further studies below.

### TMEM230 Significantly Increased Reactive Oxygen Species Level in Mitochondria and Decreased Cellular ATP Levels

**Figure 2:**
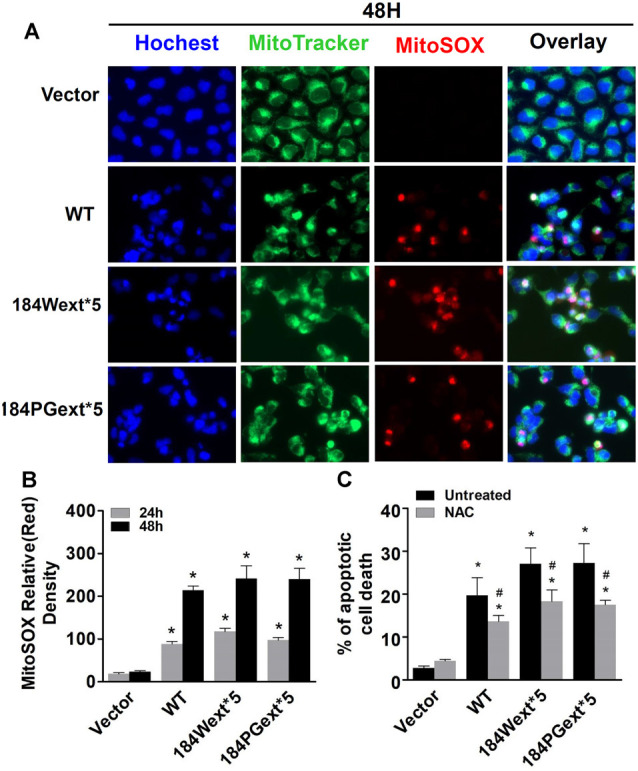
Transmembrane protein 230 (TMEM230) significantly increased reactive oxygen species (ROS) level in mitochondria. HEK293T cells were transfected with vector or various TMEM230 constructs as indicated for 24 or 48 h. MitoTracker (green) and MitoSOX (red) reagents were added to cells 5 min before image taking. **(A)** Representative cell images at 48-h post transfection. **(B)** Relative density of MitoSOX (red) fluorescence in each group at 24- and 48-h post transfection. *p* < 0.05 by ANOVA, *vs. cells expressing vector. **(C)** Cells were transfected with vector or various TMEM230 constructs as indicated for 4 h and then treated with vehicle or *N*-acetylcysteine (NAC; 1 mM) for 36 h. Apoptotic cells were counted using Hoechst 33342 staining. *p* < 0.05 by ANOVA, *vs. vector cells treated with vehicle, ^#^vs. cells expression wild-type (WT) TMEM230 variant alone.

To study whether TMEM230 alters mitochondrial reactive oxygen species (ROS), MitoSOX florescent assays were used. The MitoSOX™ Red reagent can permeate live cells where it selectively labels the production of superoxide by mitochondria under fluorescence microscopy. Expression of both WT and mutant TMEM230 increased ROS level in mitochondria in a time-dependent manner compared with that of vector cells (Figures 2A,B). Consistent with toxicity results, WT TMEM230 also increased ROS levels, whereas cells expressing TMEM230 mutants were slightly higher than those of WT TMEM230. Treatment of NAC, an ROS scavenger, reduced TMEM230-induced apoptotic cell death compared with that of untreated control cells (Figure 2C).

**Figure 3:**
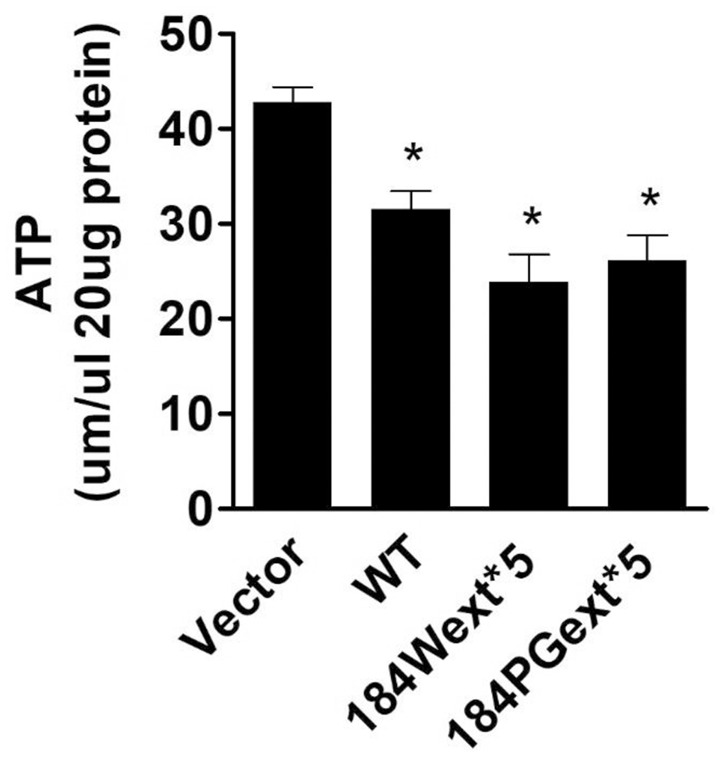
Transmembrane protein 230 (TMEM230) decreased cellular ATP. The cell lysate of HEK293T cells expressing vector or various TMEM230 constructs were subjected to ATP assays. *p* < 0.05 by ANOVA, *vs. cells expressing vector.

Increased ROS (oxidative stress) could induce mitochondrial dysfunction and result in reduction of ATP production levels (van Hameren et al., 2019). Indeed, cellular ATP levels were significantly reduced in cells expressing both WT and mutant TMEM230 compared with vector control cells (Figure 3). Expression of mutant TMEM230 decreased ATP levels severely compared with that of WT TMEM230. These results suggest that TMEM230 induced oxidative stress and mitochondrial dysfunction.

### TMEM230 Induced Caspase 3/7 Activation

**Figure 4:**
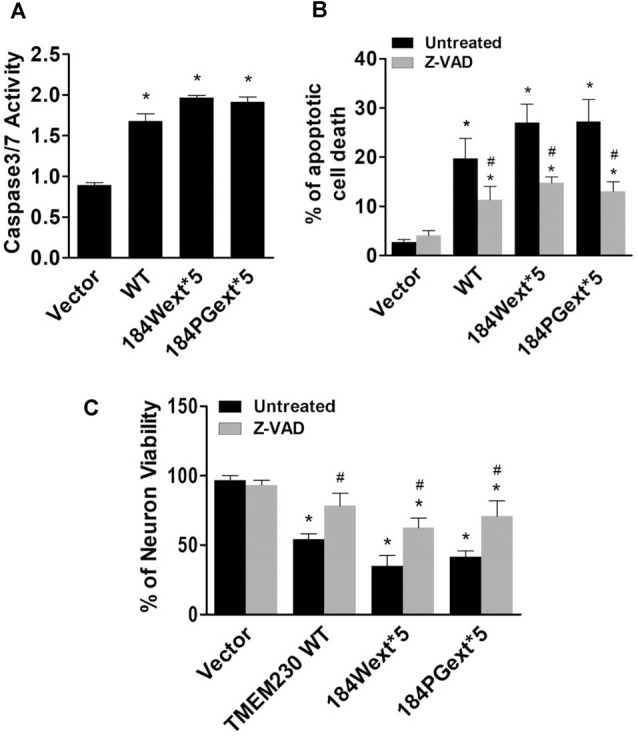
Transmembrane protein 230 (TMEM230) induced caspase 3/7 activation. **(A)** The cell lysate of HEK293T cells expressing vector or various TMEM230 constructs were subjected to caspase 3/7 activity assays. *p* < 0.05 by ANOVA, *vs. cells expressing vector. **(B)** Cells were transfected with vector or various TMEM230 constructs as indicated for 4 h and then treated with vehicle or Z-VAD-FMK (50 μM) for 36 h. Apoptotic cells were counted using Hoechst 33342 staining. **(C)** Primary neuron viability assays. *p* < 0.05 by ANOVA, *vs. vector cells treated with vehicle, ^#^vs. cells expression WT TMEM230 variant alone.

Caspase-3/7 activation is one of key features for apoptotic cell death (Fernandes-Alnemri et al., 1994; Strasser et al., 2000). We examined whether TMEM230 activates caspase 3/7 using a commercial available caspase 3/7 activity kit. Expression of both WT and mutant TMEM230 increased caspase 3/7 activation compared with that of vector cells (Figure 4A). There was an increase in caspase 3/7 activation in cells expressing mutant TMEM230 than those of WT TMEM230. Treatment of a pan caspase inhibitor, Z-VAD-FMK, at 50-μM concentration significantly protected against TMEM230-induced apoptotic cell death compared with that of untreated control cells (Figure 4B). We further validated these results using mouse cortical primary neurons. Similarly, expression of mutant TMEM230 variants significantly reduced neuronal viability compared with that of vector alone neurons (Figure 4C). The WT TMEM230 also slightly decreased the neuronal viability. Treatment of Z-VAD-FMK significantly improved the neuronal viability in neurons expressing WT and mutant TMEM230 variants compared with that of untreated neurons (Figure 4C).

### TMEM230 Induced PARP1 Cleavage

**Figure 5:**
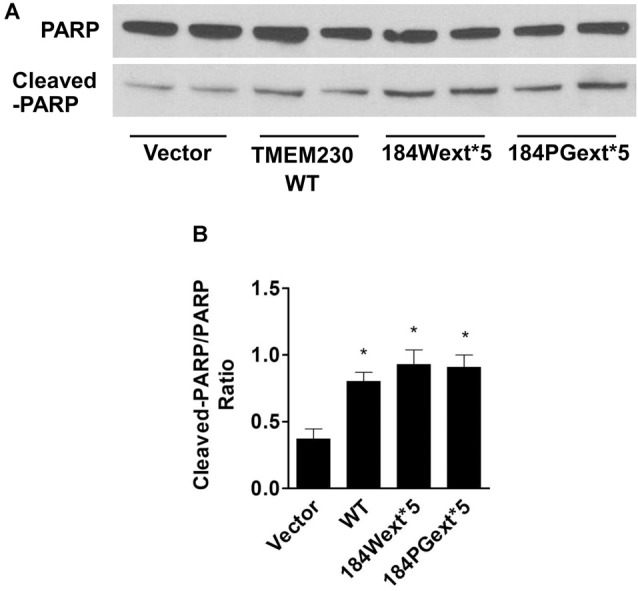
Transmembrane protein 230 (TMEM230) induced poly(ADP-ribose) polymerase (PARP) cleavage. The cell lysate of HEK293T cells expressing vector or various TMEM230 constructs was subjected to western blot analysis using anti-PARP and anti-cleaved-PARP antibodies. **(A)** Representative blot from three repeated experiments. **(B)** Quantification of **(A)** by normalization with β-actin loading control. *p* < 0.05 by ANOVA, *vs. cells expressing vector.

**Figure 6:**
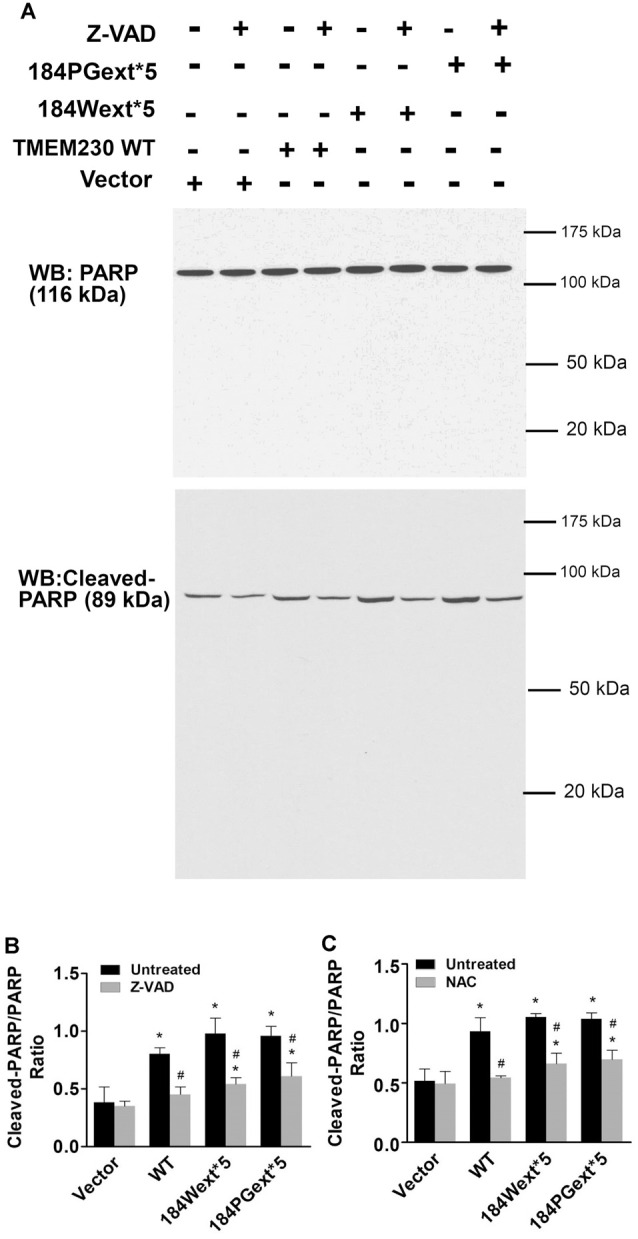
Poly(ADP-ribose) polymerase (PARP) cleavage mediated transmembrane protein 230 (TMEM230)-induced apoptotic cell death. HEK293T cells were transfected with vector or various TMEM230 constructs as indicated for 4 h and then treated with vehicle, Z-VAD-FMK (50 μM), or *NAC* (1 mM) for 36 h. Cells were harvested and subjected to western blot analysis using anti-PARP and anti-cleavage PARP antibodies. **(A)** Representative blots. **(B)** Z-VAD-FMK. **(C)** NAC. *p* < 0.05 by ANOVA, *vs. vector cells treated with vehicle, ^#^vs. cell expression WT TMEM230 variant alone.

PARP1 is an enzyme in the nucleus and plays a critical role in regulating apoptosis, DNA repair, and other cellular functions (Ke et al., 2019). PARP1 can be cleaved (inactive) by active caspase-3 during apoptosis (Chaitanya et al., 2010). We found that expression of WT and mutant TMEM230 significantly increased PARP1 cleavage (inactivation) about 2-fold of vector cells (Figure 5). Treatment with a pan caspase inhibitor, Z-VAD-FMK, at 50-μM concentration reduced PARP1 cleavage (Figures 6A,B). Moreover, treatment of NAC at 1-mM concentration also significantly protected against TMEM230-induced cell toxicity, and reduced PARP1 cleavage (Figure 6C). These results demonstrated that the ROS/caspase/PARP pathway mediated TMEM230-induced apoptosis.

## Discussion

The major findings of this study were that ectopic expression of both WT and PD-linked mutant TMEM230 induced apoptotic cell death through mitochondrial dysfunction, caspase activation, and PARP1 cleavage. PD-linked mutant TMEM230 caused cell toxicity of an increase severity than did WT TMEM230. Treatment of NAC (an ROS scavenger) or Z-VAD-FMK (a pan caspase inhibitor) reduced PARP1 cleavage and protected against TMEM230-induced apoptotic cell death. These findings demonstrated that TMEM230 plays a critical role in the ROS/caspase/PARP1-mediated cell death pathway and provided novel insight into the pathological roles of mutant TMEM230 underlying PD pathogenesis.

One of the key pathological hallmarks of PD is dopaminergic neuron loss due to neuronal death (Dauer and Przedborski, 2003). The amount of studies have demonstrated cell death induced by environmental risk factors, or genetic mutations in PD models (Michel et al., 2016). In this study, ectopic expression of PD-linked mutant TMEM230 caused apoptotic cells, as represented by an increase of condensed and fragmented nuclei, mitochondrial ROS, caspase 3/7 activation, and PARP1 cleavage. Ectopic expression of WT TMEM230 increased apoptosis as compared with that of vector cells. This suggested that WT TMEM230 levels may be tightly controlled in the physiological condition, whereas increased its expression causes cell toxicity. In the PD cases, the WT TMEM230 levels could increase in local brain areas owing to pathological conditions (e.g., abnormal protein accumulation by impaired protein degradation pathways), thereby leading to dopaminergic neuron injury or degeneration. These results are consistent with a previous study that WT TMEM230 caused cell death in SN4741 cells and that mutant TMEM230 causes more (Nam et al., 2018). Together, these findings demonstrated that TMEM230 expression level is critical in the control of caspase/PARP1-mediated cell death. The overexpression approach remains an excellent method to study the pathological roles of mutant TMEM230, although these findings still need further studies with endogenous level of TMEM230 variants in the future.

Mitochondrial dysfunction is one of the key mechanisms of neuronal degeneration underlying PD pathogenesis (Michel et al., 2016). Increased oxidative stress and mitochondrial dysfunction are present in various PD cell and animal models and in human PD postmortem brains tissues (Schapira, 2008; Blesa et al., 2015). TMEM230 is a transmembrane protein, partially colocalized with mitochondria. Our results showed that ectopic expression of TMEM230 variants increased mitochondrial ROS, thereby leading to mitochondrial dysfunction and triggering apoptotic cell death. One of the key function features of mitochondrial dysfunction is to produce less ATP. We found that expression of TMEM230 variants decreased cellular ATP levels as compared with that of vector cells, suggesting that TMEM230 plays critical role in mitochondrial normal function. The other two PD-linked proteins, PINK1 and parkin, are resident proteins in mitochondria, in which mutations caused familial PD and mitochondrial dysfunction (Subramaniam and Chesselet, 2013). Thus, TMEM230 could potentially interplay with PINK1 and parkin underlying mitochondrial dysfunction and neurodegeneration, although this remains to be further investigated.

Mitochondrial dysfunction often activates caspase cascade thereby leading to apoptosis (Vakifahmetoglu-Norberg et al., 2017). We found that expression of TMEM230 variants significantly induced caspase 3/7 activation, which are execution caspases. Activation of caspase 3/7 could cause PARP1 cleavage to produce 89 and 24 kD, two fragments resulting in induced apoptosis (Lazebnik et al., 1994). PARP1, a nuclear enzyme, plays a critical role in regulating DNA repair and apoptosis (de Murcia et al., 1997; Chaitanya et al., 2010; Morales et al., 2014; Eisemann and Pascal, 2020). In the line with these previous findings, our results showed that expression of TMEM230 variants induced PARP1 cleavage with an antibody against 89-kD fragments. Treatment of pan caspase inhibitor, Z-VAD-FMK, or NAC (an ROS scavenger) attenuated the PARP1 cleavage and protected against TMEM230-induce toxicity. These results suggest that the ROS/caspase/PARP mediated TMEM230-induced apoptotic cell death pathway.

In conclusion, our findings demonstrated that ectopic expression of both WT and PD-linked mutant TMEM230 induced apoptotic cell death though mitochondrial dysfunction, caspase activation, and PARP1 cleavage. This is the first report that TMEM230 plays a critical role in the caspase/PARP1-mediated cell death pathway and provides the novel insight into the pathological roles of mutant TMEM230 underlying PD pathogenesis.

## Data Availability Statement

All datasets presented in this study are included in the article.

## Ethics Statement

The animal study was reviewed and approved by the Institutional Animal Care and Use Committees of the Johns Hopkins Medicine Institute.

## Author Contributions

XW, CL, and WS conceived and designed the study. XW, TW, JZ, GG, XH, CR, and ZL acquired, analyzed, and interpreted the data. TW and ZP contributed research agents. XW, CL, and WS drafted the manuscript. XW, CL, and WS performed statistical analysis.

## Conflict of Interest

The authors declare that the research was conducted in the absence of any commercial or financial relationships that could be construed as a potential conflict of interest.
